# Combined inhibition of the complement component C5 and the TLR-coreceptor CD14 alters the posttraumatic response in fracture hematoma in a porcine polytrauma model

**DOI:** 10.1515/iss-2025-0031

**Published:** 2025-11-04

**Authors:** Dong Chen, Jasper Nies, Klemens Horst, Ümit Mert, Johannes Greven, Felix Bläsius, Tom Eirik Mollnes, Markus Huber-Lang, Frank Hildebrand, Martijn van Griensven, Rald V.M. Groven

**Affiliations:** Experimental Orthopedic and Trauma Surgery, Department of Orthopedic, Trauma- and Reconstructive Surgery, RWTH Aachen University Hospital, Aachen, Germany; Department of Orthopedic, Trauma- and Reconstructive Surgery, RWTH Aachen University Hospital, Aachen, Germany; Research Laboratory, Nordland Hospital Bodø, Bodø, Norway; Department of Immunology, Oslo University Hospital, and University of Oslo, Oslo, Norway; Institute of Clinical and Experimental Trauma Immunology, University Hospital Ulm, Ulm, Germany; Department of Cell Biology-Inspired Tissue Engineering, MERLN Institute for Technology-Inspired Regenerative Medicine, Maastricht University, Maastricht, The Netherlands

**Keywords:** polytrauma, fracture hematoma, systemic inflammation, fracture healing, immunomodulation

## Abstract

**Objectives:**

Polytrauma is characterized by high mortality and morbidity rates, partly due to the post-traumatic immune response. This follow-up study investigated the effect of combined inhibition of complement factor 5 (C5) and CD14 on systemic (plasma) and local (fracture hematoma) protein levels in a porcine polytrauma model.

**Methods:**

18 male pigs (*sus scrofa*) were used for this study: a control group (n=6), and two groups that were subjected to standardized polytrauma, followed by standard treatment (intramedullary nailing; n=8) or standard treatment with C5/CD14 inhibition therapy (n=4). Plasma and fracture hematoma samples were collected at specified time points and the expression levels of proteins related to the post-traumatic immune response and fracture healing were determined using enzyme-linked immunosorbent assays.

**Results:**

Pro-inflammatory proteins such as IL-1β, IL-6, and IFN-α were significantly reduced in both plasma and fracture hematoma samples at 72 h after trauma in the treated vs. the non-treated group. Soluble TLR4, a possible inhibitor of cell membrane TLR4, was increased in both the plasma and the fracture hematoma following therapy, suggesting that TLR4 was released from the cell membrane to the fluid phase.

**Conclusions:**

These findings show that systemic C5/CD14 inhibition effectively reduced the concentration of several pro-inflammatory proteins in the systemic circulation and locally, at the fracture site, in the fracture hematoma. The levels in the plasma were in line with those in the fracture hematoma, albeit that they were markedly lower in the plasma compared to the fracture hematoma.

## Introduction

Polytrauma is associated with high mortality and morbidity rates, in part because of the post-traumatic immune response [1], [2], [3], [4]. Directly after trauma, the innate immune system is activated, attracting various immune cells such as neutrophils and macrophages to the sites of injury which, under physiological circumstances, guide tissue clearance and regeneration [5]. However, polytrauma can exacerbate this immune response leading to systemic and local pathophysiological inflammation. This can subsequently lead to organ dysfunction and reduced tissue regenerative capacities at for instance fracture sites [6], 7].

Adequate fracture healing depends on various cell types and molecular processes. Immediately after a fracture occurs, a hematoma is formed at the fracture site in which pro-inflammatory mediators and various growth factors are released, which are key for the chemoattraction of immune cells and the initiation and regulation of the fracture healing process [8]. Furthermore, this hematoma also serves as a scaffold for fibroblasts and mesenchymal stem cells (MSCs), which are vital for the formation of a callus and its later mineralization. Research has shown that exacerbated immune responses after polytrauma are associated with impaired fracture healing due to an immunological imbalance at the fracture site, contributing to prolonged patient immobilization and recovery [8], 9].

Taken together, modulation of the immune response after polytrauma is of clinical relevance to reduce associated organ dysfunction in the acute phase after trauma, whilst also promoting tissue regeneration. As critical parts of the early innate immune response, toll-like receptor (TLR) signaling and the complement system are promising therapeutic targets [10], [11], [12], [13]. More specifically, an inhibition of complement factor 5 (C5), leading to a partial blockade of the complement system, reduces the attraction of immune cells and the subsequent inflammatory response by preventing the release of the potent anaphylatoxin C5a, and in addition prevents the formation of membrane attack complexes [14], 15]. Furthermore, by blocking both TLR4 and TLR2 signaling at the TLR co-receptor CD14 level in the acute phase after polytrauma, damage- and pathogen-associated molecular pattern recognition is decreased [16]. Apart from *in vivo* murine sepsis models in which such a combined blockade of C5 and CD14 (C5/CD14) has shown promising results, research has shown that the application of C5/CD14 inhibition in the acute phase after polytrauma successfully reduced catecholamine requirement, systemic inflammation, and end-organ damage in a porcine model of polytrauma [17], [18], [19], [20]. However, research on the effect of such systemically applied immunomodulatory therapy on local tissue responses at the fracture site, is lacking.

Therefore, we defined two follow-up study aims from data and materials available from the original study [17], comparing two treatment groups in a porcine polytrauma model: 1) to investigate potential associations between systemic (plasma) and local (fracture hematoma) protein patterns, and 2) to investigate the effect of inhibiting TLR signaling and the complement system (C5/CD14 inhibition) on the expression of these proteins, both in the systemic circulation and locally, at the fracture site.

## Materials and methods

### Animals

The data presented in this paper were collected as a follow-up study in the context of a larger collaborative study [17] in accordance with the principles of refining, reducing, and replacing the use of animals in laboratory research (3R principles). The present study was determined *a priori* and the data presented are original and reported in adherence to the ARRIVE guidelines. The German governmental office of animal care and use approved the protocols and procedures of this study (permit 81-02.04.2020.A215).

In total, 18 male pigs (*sus scrofa*; German Landrace) were used with an age of three months and a mean body weight of 35 ± 5 kg. Upon arrival, all animals were examined by a veterinarian and housed for seven days prior to the start of the experiment for acclimatization purposes.

### Interventional drugs

RA101295 (2 kDa peptide), a pan-species C5 inhibitor, was provided by UCB Pharma (Brussels, Belgium). It inhibits the proteolytic cleavage of C5, blocking the formation of the anaphylatoxin C5a and the terminal complement complex (TCC), which manifests as either soluble C5b-9 (sC5b-9) or as membrane attack complex (MAC) in the cell wall.

rMil2 is a recombinant CD14 antibody (porcine specific, clone MIL2; isotype IgG2a) produced as an IgG2/4 chimera in the laboratory of Professor T. E. Mollnes (Norway) and on a large scale by ExcellGene SA (Monthey, Switzerland) according to GMP standards [21].

### General instrumentation and anesthesia

**Figure 1:**
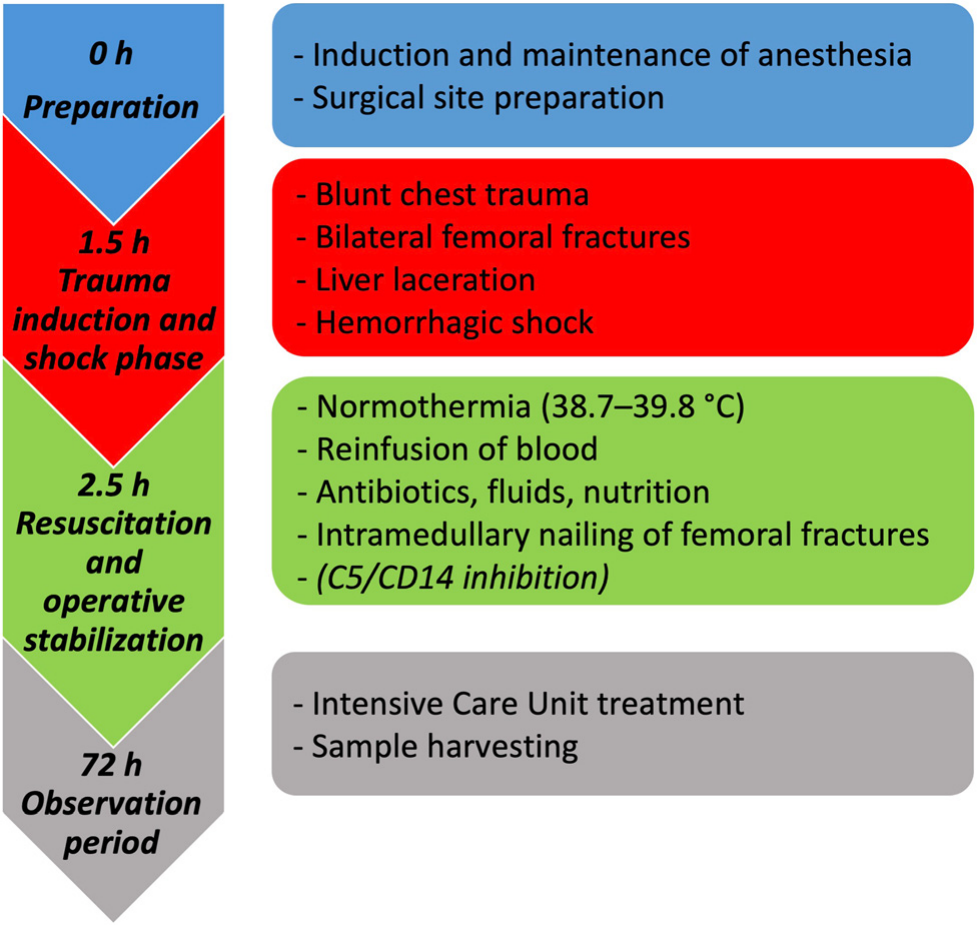
Schematic overview of the experimental workflow.

The porcine polytrauma model was established by our group and has been described in extensive detail before [17], 19]. A schematic overview of the experimental workflow is depicted in Figure 1. In short, after a 12-h fasting period with unrestricted access to water, animals were premedicated with the intramuscular injection of azaperone (Stresnil™, Janssen-Cilag GmbH, Neuss, Germany) and ketamine (Ketanest, Pfizer, NY). General anesthesia was induced using propofol (Fresenius SE & Co. KGaA, Bad Homburg, Germany) after which orotracheal intubation was performed, volume-controlled mechanical ventilation was started (tidal volume of 8–12 mL/kgBW, peak end-expiratory pressure of 8 mmHg, and a plateau pressure <28 mmHg), and continuous monitoring of vital signs was established, including the placement of arterial and central venous access for the continuous invasive monitoring of blood pressure, execution of the hemorrhagic shock, and administration of fluids, nutrition, and medicaments, respectively [22]. Animals were kept under general anesthesia using propofol (Fresenius SE & Co. KGaA), fentanyl (Panpharma GmbH, Trittau, Germany), and midazolam (Panpharma GmbH) throughout the entire experiment. Three experimental groups were defined: control (n=6), polytrauma (PT; n=8), and PT receiving C5 and CD14 inhibiting therapy (PT+C5/CD14; n=4).

### Induction of trauma and hemorrhage

All groups received identical instrumentation, anesthesia, and parental nutrition. The PT and PT+C5/CD14 groups were subsequently exposed to a standardized polytrauma (injury severity score=27) [22]. Stable baseline conditions for 120 min were a prerequisite for starting trauma induction and hemorrhagic shock. The fraction of inspired oxygen was set to 0.21 throughout the trauma and shock phases to simulate ambient air conditions. Pigs were then first exposed to blunt chest trauma using a bolt gun machine (turbocut Jopp GmbH, Bad Neustadt, Germany), hitting a set of metal panels (steel 8 mm, lead 10 mm) on the right caudal dorsal chest. Second, bilateral femoral fractures were established using the bolt gun machine and a metal punch, placed on the medial part of the femur shaft. Third, via a midline laparotomy, the left liver lobe was exposed and a crosswise incision (4.5 × 4.5 cm; halfway through the liver parenchyma) was made to mimic liver laceration and left to bleed uncontrolled for 30 s prior to standard packing. Fourth, hemorrhagic shock was established (mean arterial pressure 40 ± 5 mmHg or maximally withdrawing 45 % of the total blood volume). After completion of the four components of the polytrauma, animals were left untreated for 90 min to imitate a pre-clinical shock phase; no fluids were administered, and hypothermia was not prevented.

### Resuscitation and operative stabilization

After the 90 min shock phase, animals were resuscitated according to current trauma guidelines (ATLS^®^, AWMF-S3 guideline on Treatment of Patients with Severe and Multiple Injuries^®^) [23]. Normothermia was maintained with blankets and air blower systems. All withdrawn blood was re-infused (Citrate Phosphate Dextrose Adenine DONOpacks, Lmb Technologie GmbH, Oberding, Germany) in conjunction with crystalloid fluids (Sterofundin ISO^®^), and ventilatory settings were adjusted based on arterial blood gas analyses.

Bilateral femoral fractures were stabilized via intramedullary nailing (T2 System, Stryker GmbH & Co. KG, Duisburg, Germany). Before surgery and every 24 h after trauma induction, 2 g of ceftriaxone (Fresenius SE & Co. KGaA, Homburg, Germany) were administered. Parenteral nutrition (Aminoven, Fresenius Kabi Deutschland GmbH, Bad Homburg, Germany) and crystalloids were provided under careful observation of blood glucose levels and the fluid balance.

One animal in the treatment group received a bolus of C5 inhibitor (3 mg/kg) and anti-CD14 (5 mg/kg) at 30 min post-injury, followed by continuous C5 inhibitor infusion (0.55 mg/kg/h) until 64 h. Based on the obtained results, the remaining three animals received increased doses: a C5 inhibitor bolus (5 mg/kg) at 30 min post-injury, followed by infusion (1.1 mg/kg/h) until 72 h, and anti-CD14 boluses of 5 mg/kg at 30 min, 12, and 30 h, and 2.5 mg/kg at 60 h. All four animals were analyzed as a single group due to minimal variability in treatment efficacy (p<0.05 for all parameters comparing 3 vs. 8 and 4 vs. 8 animals).

### Sample and data collection

Whole blood samples were obtained at 2.5 and 72 h after trauma and stored in ethylenediaminetetraacetic acid tubes (S-Monovette, Sarstedt, Nümbrecht, Germany) which were kept on ice. Subsequently, the whole blood samples were centrifuged at 2,000×*g* for 15 min at a temperature of 4 °C. The plasma was pipetted of the sample and stored at −80 °C for further analysis. Fracture hematoma samples were obtained from the fracture site at 72 h after trauma in the PT and the PT+C5/CD14 inhibition group, and stored at −80 °C. Enzyme-linked immunosorbent assays for VEGF-C (Antibodies-online, Philadelphia, PA, USA), FLT3LG (Lsbio, Seattle, USA), TLR4 (Mybiosource, San Diego, USA), IFN-α, IL-1β, IL-6, IL-8, IL-10, and IL-12/IL-23 p40 (Lsbio, Seattle, USA) were used according to the manufacturer’s instructions.

### Statistical analysis

Statistical analyses were performed using GraphPad Prism (version 9.1.1, GraphPad Software Inc., San Diego, CA). The Kolmogorov–Smirnov test was used to test for normality, after which a two-way ANOVA was used to determine differences within time points between the groups. All data are expressed as the mean accompanied by the standard error of the mean (mean ± SEM). An α of 0.05 was considered statistically significant.

## Results

The present follow-up analysis included a total of 18 animals [17]. One animal in the PT group died at 60 h after trauma, most probably due to cardiorespiratory failure, and was excluded from the analyses.

### Plasma protein expression

**Figure 2:**
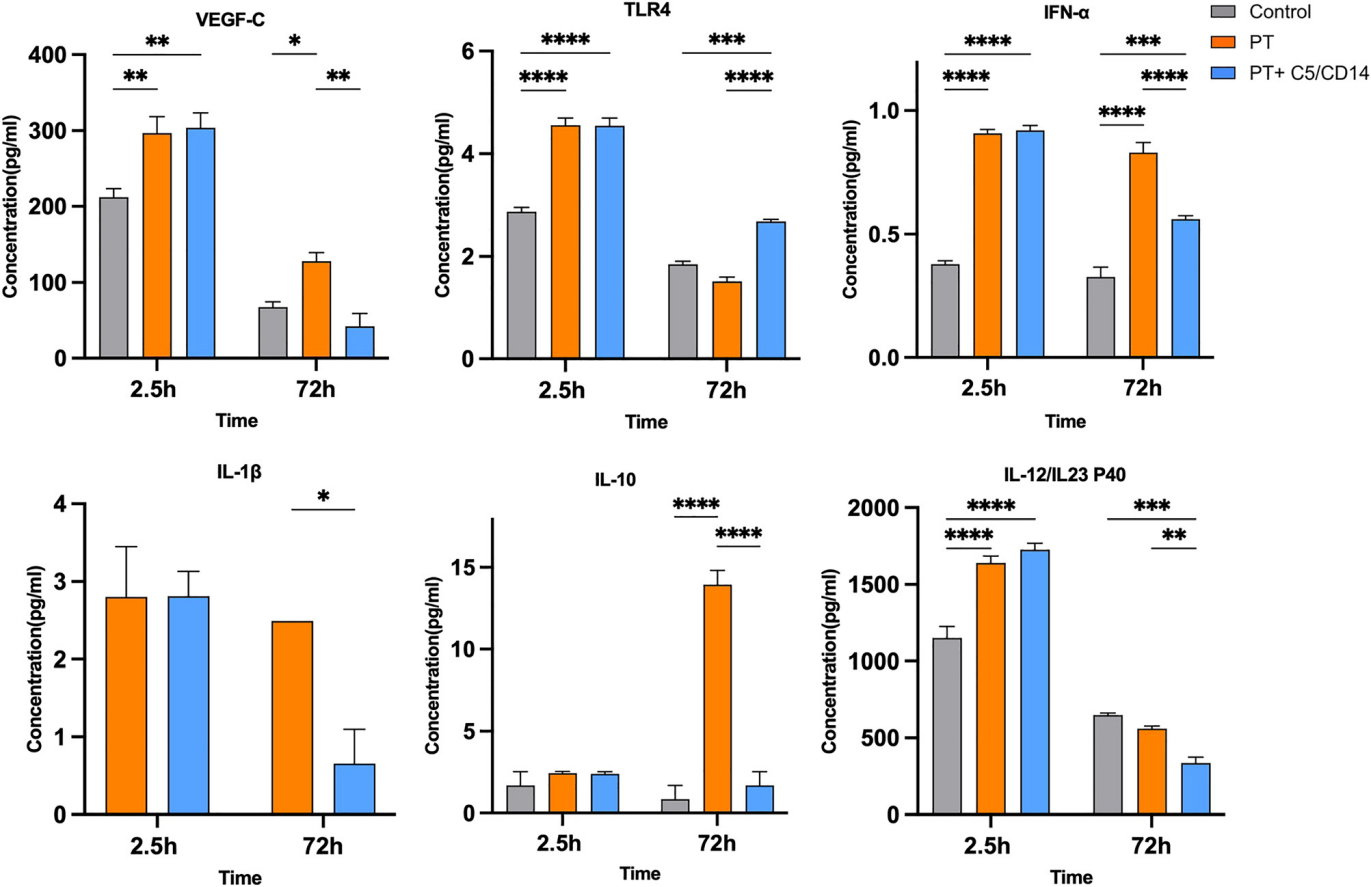
Protein concentrations at 2.5 and 72 h after trauma in plasma samples from the control, polytrauma (PT), and PT groups receiving C5/CD14 inhibition therapy (PT+C5/CD14). VEGF-C, vascular endothelial growth factor C; sTLR4, soluble toll like receptor 4; IFN-α, interferon alpha; IL-1β, interleukin-1 beta; IL-10, interleukin-10; IL-12/IL23 P40, interleukin-12/interleukin23 P40; pg/mL, picogram per milliliter; *p≤0.05; **p≤0.01; ***p≤0.001; ****p≤0.0001. Results are depicted as bars, error bars indicate the standard error of the mean. The expression of IL-1β was undetectable in the control group at 2.5 and 72 h.

Plasma protein concentrations were determined in the early acute post-traumatic phase at 2.5 and 72 h after trauma. At 2.5 h after trauma, the plasma concentrations of VEGF-C, soluble TLR4 (sTLR4), IFN-α, IL-1β, and IL-12/IL-23 p40 (all p<0.05) all resulted significantly higher in both the PT and PT+C5/CD14 inhibition group compared to the control group. For plasma IL-10 concentration, no differences were observed among the groups, indicating that its systemic expression in the acute post-traumatic phase is not influenced by either trauma or the C5/CD14 inhibition therapy. Furthermore, at the 2.5 h time point, no significant differences in plasma protein concentrations between the PT and PT+C5/CD14 inhibition groups were observed. At 72 h after trauma, sTLR4 was the only protein whose concentration increased in the PT+C5/CD14 inhibition group relative to the PT group (p<0.001). The plasma concentrations of VEGF-C (p=0.006), IFN-α (p<0.001), IL-1β (p=0.03), IL-10 (p<0.001), and IL-12/IL-23 p40 (p=0.009) all decreased significantly compared to the PT group (Figure 2). The plasma concentrations of FLT3LG and IL-8 fell below the detection range.

### Fracture hematoma protein expression

**Figure 3:**
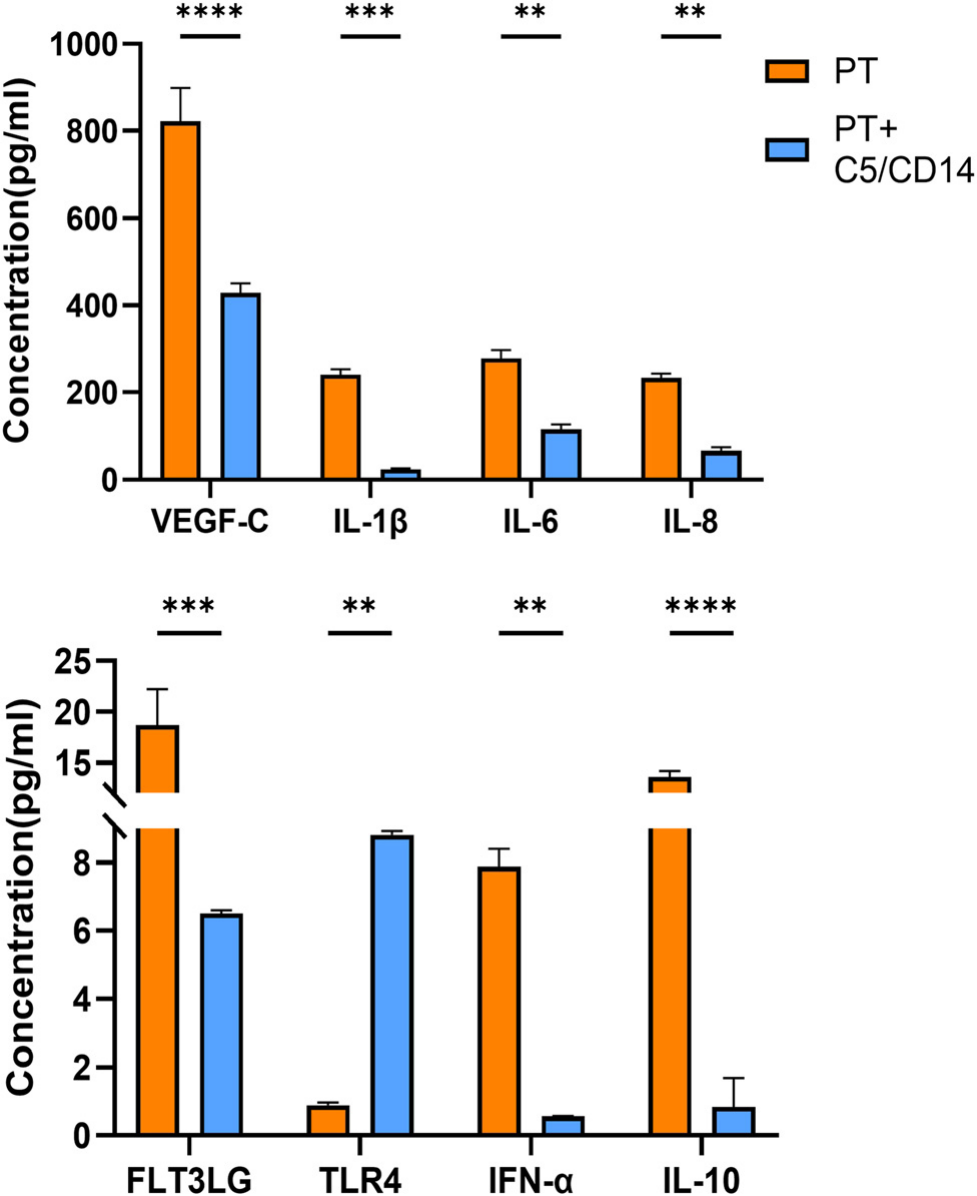
Protein concentrations at 72 h after trauma in fracture hematoma samples from the polytrauma (PT), and PT groups receiving C5/CD14 inhibition therapy (PT+C5/CD14). VEGF-C, vascular endothelial growth factor c; IL-1β, interleukin-1 beta; IL-6, interleukin-6; IL-8, interleukin-8; FLT3LG, Fms-related tyrosine kinase 3 ligand; TLR4, toll like receptor 4; IFN-α, interferon alpha; IL-10, interleukin-10. pg/mL, picogram per milliliter; **p≤0.01; ***p≤0.001; ****p≤0.0001. Results are depicted as bars, error bars indicate the standard error of the mean.

In the fracture hematoma, protein concentrations were measured at 72 h after trauma. The concentrations of VEGF-C (p<0.0001), FLT3LG (p=0.0002), IFN-α (p=0.0070), IL-1β (p=0.0004), IL-6 (p=0.0055), IL-8 (p=0.0046), and IL-10 (p<0.0001) were all significantly lower in the PT+C5/CD14 inhibition group compared with the PT group. Conversely, the fracture hematoma concentration of TLR4 (p=0.0037) increased significantly in the PT+C5/CD14 inhibition group in comparison to the PT group (Figure 3). The concentrations of IL-12/IL-23 p40 fell below the detection range.

## Discussion

Following trauma, the activation of the innate immune system is a delicate balance, as excessive complement activation contributes to post-traumatic inflammatory complications, while insufficient activation hinders adequate tissue regeneration [24], 25]. Increased systemic inflammation and persistent innate inflammatory stimuli in part underlie impaired tissue regeneration, such as fracture healing disturbances after polytrauma [23], 26]. Therefore, this study investigated the therapeutic potential of C5/CD14 inhibition therapy in restoring immunological homeostasis in the systemic circulation and in the fracture hematoma during the acute phase after trauma. The main findings of the study can be summarized as follows:

- In the acute post-traumatic phase, the expression of pro-inflammatory cytokines (IL-1β, IFN-α, IL-12/IL-23 P40) was significantly reduced at 72 h in both the plasma and fracture hematoma of the C5/CD14 inhibition group.
- Soluble TLR4 increased in both the plasma and fracture hematoma in the C5/CD14 inhibition group, suggesting the release of the TLR4 receptor from the surface of the cells, lowering the cell response to TLR4 ligands, further supporting the effect on the inflammatory response of the inhibition.
- The protein concentrations in the plasma matched with those in the fracture hematoma, albeit that they were markedly lower in the plasma compared to the fracture hematoma.

These observations are supported by previous research in pre-clinical and clinical settings. Work by Sommerfeld *et al.* showed that inhibiting C5a in a murine sepsis model decreased mortality and also reduced plasma IL-10 expression, as in the present study [27]. Similarly, Yang *et al.* reported that a systemically applied C5 inhibitor in a rat model of blast trauma and hemorrhagic shock attenuated the expression of pro-inflammatory cytokines IL-6, TNF-α, and IL-1β in injured tissues, which is in line with our findings in the fracture hematoma [28]. Similarly, results from a long-term non-human primate sepsis model showed that C5 inhibition reduced mortality and organ failure and also significantly decreased the systemic expression of IL-6, IL-8, and TNF-α over time [29].

Inhibition of CD14 blocks ligands to both TLR2 and TLR4, the latter being an important mediator in the humoral and cellular immune response [16]. LPS is the main ligand for binding to TLR4 and subsequent signaling. Keshari *et al.* demonstrated that CD14 inhibition improved survival and reduced thrombo-inflammation in a baboon sepsis model by decreasing the activation of inflammatory cells, such as neutrophils, and a significant reduction in the expression of systemic IL-6, IL-1β, and IL-8 [30]. Similar findings were observed by Olszyna *et al.*, who showed that CD14 inhibition in human endotoxemia effectively reduced the systemic expression of the pro-inflammatory cytokine IL-8 [31]. Taken together, our results are in line with those of the abovementioned studies showing the therapeutic potential of CD14 inhibition in reducing systemic inflammation. Interestingly, despite the overall suppression of inflammatory cytokines, we observed an increased level of soluble TLR4 in the plasma and the fracture hematoma of the C5/CD14 inhibition group at 72 h after trauma. This suggests a possible release of the membrane TLR4 receptor to the fluid phase as sTLR4, then lowering the cells’ potential to be activated through TLR4.

While combined C5/CD14 inhibition therapy has shown promising results in sepsis, little research focused on its potential application in the polytrauma setting so far [18], [32], [33], [34]. Prior work by our group demonstrated that C5/CD14 inhibition therapy decreased organ failure while also reducing vasopressor requirements in a porcine model of polytrauma [17]. However, for both C5 and CD14 inhibition therapy, long-term effects on fracture healing outcome are not known and remain a topic of ongoing research [35]. For C5 inhibition, few case reports exist on patients suffering from osteochondritis and joint abnormalities after long-term C5 inhibition therapy, for e.g. factor H deficiency [36]. On the other hand, other studies demonstrated a therapeutic potential of a C5 inhibition for conditions like rheumatoid arthritis and osteoporosis [37]. For CD14, there is increasing evidence suggesting CD14 plays an important role in wound healing and angiogenesis. However, no negative effects of CD14 inhibition therapy on fracture healing or bone regeneration have been observed until present [38], 39].

Immune modulation after trauma is a dynamic field of ongoing research in which the innate immune system plays an important role. This study is among the first to illustrate that systemically applied C5/CD14 inhibition therapy can modulate the inflammatory microenvironment at the fracture site in the acute post-traumatic phase.

A limitation of this study is the duration of the model, which is too short to study the long-term outcome of the C5/CD14 inhibition on fracture healing. Furthermore, a topic of further research should be the temporal changes in cytokine expression in the fracture hematoma following C5/CD14 inhibition therapy; this was not possible in the present study due to its design. Both further *in vitro* as well as *in vivo* experiments would be suitable in the exploration of long-term effects of the C5/CD14 inhibition therapy. In the *in vitro* setting, the C5/CD14 inhibition therapy could be applied in novel three-dimensional cell cultures, such as organ-on-a-chip or organoid models, to extensively investigate the long-term effect of the applied therapy on specific organ functions such as the fracture site or the lungs. However, to validate *in vitro* findings, a long-term (e.g. two weeks after trauma) *in vivo* model is required to explore the effect of the C5/CD14 inhibition therapy at the individual organ level but also to investigate its role and involvement in organ crosstalk over time after trauma.

## Conclusions

The present study showed that systemic C5/CD14 inhibition effectively reduced the concentration of several pro-inflammatory proteins in the systemic circulation and locally, at the fracture site, in the fracture hematoma. The levels in the plasma were in line with those in the fracture hematoma, albeit that they were markedly lower in the plasma compared to the fracture hematoma.
